# *catena*-Poly[[(nitrato-κ^2^ *O*,*O*′)silver(I)]-μ-1,2-bis­(diphenyl­phosphino)ethane-κ^2^ *P*:*P*′]

**DOI:** 10.1107/S1600536808019442

**Published:** 2008-07-05

**Authors:** Xing-Cong Wang, Yan-Li Wu, Xiu-Li You

**Affiliations:** a Jiangxi Key Laboratory of Organic Chemistry, Jiangxi Science and Technology Normal University, Nanchang 330013, People’s Republic of China

## Abstract

In the title chain compound, [Ag(NO_3_)(C_26_H_24_P_2_)]_*n*_, the bis­(diphenyl­phosphino)ethane (dppe) units link the Ag^+^ ions into chains along [001]. A nitrate anion is coordinated to the Ag atom. There is a centre of symmetry at the mid-point of the ethane C—C bond and a twofold rotation axis passes through the Ag, N and terminal O atoms. Each Ag atom is four-coordinated in a distorted tetra­hedral geometry by two O atoms of the nitrate anion and two P atoms of dppe ligands. The two aromatic rings are oriented at a dihedral angle of 73.77 (3)°.

## Related literature

For related literature, see: Harker & Tiekink (1990 ▶); Huang *et al.* (1991 ▶); Menezes Vicenti & Burrow (2007); Yang *et al.* (1992 ▶).
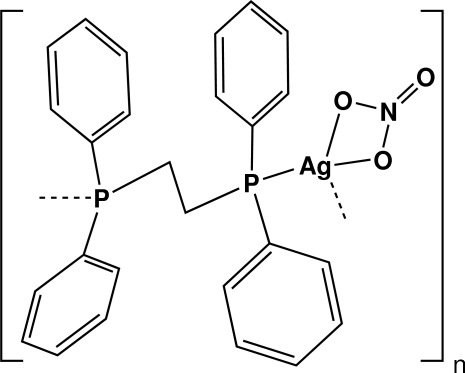


## Experimental

### 

#### Crystal data

[Ag(NO_3_)(C_26_H_24_P_2_)]
*M* *_r_* = 568.27
Monoclinic, 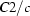

*a* = 17.123 (3) Å
*b* = 14.064 (3) Å
*c* = 11.120 (2) Å
β = 108.33 (3)°
*V* = 2542.0 (9) Å^3^
*Z* = 4
Mo *K*α radiation
μ = 0.95 mm^−1^
*T* = 223.2 K
0.30 × 0.26 × 0.20 mm

#### Data collection

Rigaku Mercury diffractometer
Absorption correction: multi-scan (Jacobson, 1998 ▶) *T*_min_ = 0.704, *T*_max_ = 0.833
12170 measured reflections
2327 independent reflections
2161 reflections with *I* > 2σ(*I*)
*R*_int_ = 0.030

#### Refinement

*R*[*F* ^2^ > 2σ(*F* ^2^)] = 0.035
*wR*(*F* ^2^) = 0.087
*S* = 1.07
2327 reflections
151 parameters
H-atom parameters constrained
Δρ_max_ = 0.97 e Å^−3^
Δρ_min_ = −0.45 e Å^−3^

### 

Data collection: *CrystalClear* (Rigaku/MSC, 2001 ▶); cell refinement: *CrystalClear*; data reduction: *CrystalStructure* (Rigaku/MSC, 2004 ▶); program(s) used to solve structure: *SHELXS97* (Sheldrick, 2008 ▶); program(s) used to refine structure: *SHELXL97* (Sheldrick, 2008 ▶); molecular graphics: *SHELXL97*; software used to prepare material for publication: *SHELXL97*.

## Supplementary Material

Crystal structure: contains datablocks I, global. DOI: 10.1107/S1600536808019442/hk2480sup1.cif

Structure factors: contains datablocks I. DOI: 10.1107/S1600536808019442/hk2480Isup2.hkl

Additional supplementary materials:  crystallographic information; 3D view; checkCIF report

## Figures and Tables

**Table:** 

Ag1—P1	2.4066 (9)
Ag1—O1	2.508 (2)

**Table:** 

P1—Ag1—P1^i^	137.49 (4)
P1—Ag1—O1^i^	115.92 (7)
P1—Ag1—O1	102.63 (6)
O1^i^—Ag1—O1	50.52 (11)

Symmetry code: (i) 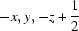.

**Fig. 1.**
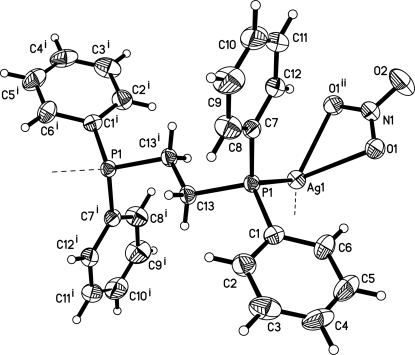
The molecular structure of the title molecule, with the atom-numbering scheme. Displacement ellipsoids are drawn at the 50% probability level [symmetry codes: (i) -x, 1 - y, -z; (ii) -x, y, 1/2 - z].

**Fig. 2.**
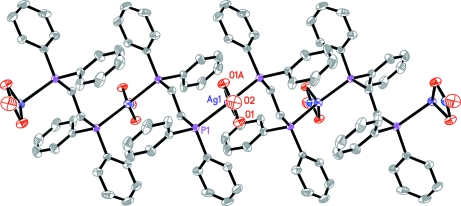
The coordination polymer of (I) [symmetry code: (A) -x, y, 1/2 - z] along the c axis.

## supplementary crystallographic information

### Comment

The complexes obtained by the reaction of AgNO_3_ with bis(diphenylphosphino)- ethane), dppe, include mono-nuclear complex Ag(dppe)(NO_3_), (II) (Harker & Tiekink, 1990), binuclear complex [Ag(dppe)]_2_(NO_3_)_2_.2MeOH, (III) (Yang *et al.*,  1992), and one-dimensional polymers: [Ag_4_(dppe)_3_(NO_3_)_4_]_n_, (III) (Huang *et al.*,  1991) and [Ag(dppe)(NO_3_)(DMF)]_n_, (IV) (Menezes Vicenti & Burrow, 2007). We report herein the formation of a one-dimensional coordination polymer, (I), using AgNO_3_ as the metal source and dppe as bidentate bridging ligand, and its crystal structure.

The structure of the title compound, (I), is polymeric with dppe bridging ligands between Ag centres to form a chain (Fig. 1). There is one dppe ligand in the asymmetric unit. The remaining parts are generated by crystallographic centres of inversion at the mid-points of the C-C bond of the ethane group. The polymeric chains are elongated along [001] direction (Fig. 2). A nitrate anion is coordinated to the Ag atom, in which a twofold rotation axis passes through the N1-O2 bond. Each Ag atom is four-coordinated in a distorted tetrahedral geometry (Table 1) by two O atoms of the nitrate anion and two P atoms of dppe ligands. The two aromatic rings are oriented at a dihedral angle of 73.77 (3)°.

### Experimental

For the preparation of the title compound, dppe (20 mg, 0.05 mmol) was dissolved in CH_2_Cl_2_ (5 ml) and was poured into the tube, then MeOH (3 ml) was layered on it. Finally, MeOH solution (5 ml) containing AgNO_3_ (8.5 mg, 0.05 mmol) was layered on the top of the tube. The crystals of the title compound, (I), were obtained for about 3 d.

### Refinement

H atoms were positioned geometrically, with C-H = 0.93 and 0.97 Å for aromatic and methylene H and constrained to ride on their parent atoms with U_iso_(H) = 1.2U_eq_(C).

### Crystal data

**Table:**

[Ag(NO_3_)(C_26_H_24_P_2_)]	*F*_000_ = 1152
*M**_r_* = 568.27	*D*_x_ = 1.485 Mg m^−^^3^
Monoclinic, *C*2/*c*	Mo *K*α radiation λ = 0.71073 Å
Hall symbol: -C 2yc	Cell parameters from 5049 reflections
*a* = 17.123 (3) Å	θ = 3.5–25.3º
*b* = 14.064 (3) Å	µ = 0.95 mm^−^^1^
*c* = 11.120 (2) Å	*T* = 223.2 K
β = 108.33 (3)º	Block, colorless
*V* = 2542.0 (9) Å^3^	0.30 × 0.26 × 0.20 mm
*Z* = 4	

### Data collection

**Table:**

Rigaku Mercury diffractometer	2327 independent reflections
Radiation source: fine-focus sealed tube	2161 reflections with *I* > 2σ(*I*)
Monochromator: graphite	*R*_int_ = 0.031
Detector resolution: 14.6306 pixels mm^-1^	θ_max_ = 25.3º
*T* = 223.15 K	θ_min_ = 3.5º
ω scans	*h* = −20→19
Absorption correction: multi-scan(Jacobson, 1998)	*k* = −16→15
*T*_min_ = 0.704, *T*_max_ = 0.833	*l* = −13→13
12170 measured reflections	

### Refinement

**Table:**

Refinement on *F*^2^	Secondary atom site location: difference Fourier map
Least-squares matrix: full	Hydrogen site location: inferred from neighbouring sites
*R*[*F*^2^ > 2σ(*F*^2^)] = 0.035	H-atom parameters constrained
*wR*(*F*^2^) = 0.087	*w* = 1/[σ^2^(*F*_o_^2^) + (0.0457*P*)^2^ + 4.2828*P*] where *P* = (*F*_o_^2^ + 2*F*_c_^2^)/3
*S* = 1.07	(Δ/σ)_max_ < 0.001
2327 reflections	Δρ_max_ = 0.97 e Å^−^^3^
151 parameters	Δρ_min_ = −0.45 e Å^−^^3^
Primary atom site location: structure-invariant direct methods	Extinction correction: none

### Special details

**Table:**

Experimental. no
Geometry. All e.s.d.'s (except the e.s.d. in the dihedral angle between two l.s. planes) are estimated using the full covariance matrix. The cell e.s.d.'s are taken into account individually in the estimation of e.s.d.'s in distances, angles and torsion angles; correlations between e.s.d.'s in cell parameters are only used when they are defined by crystal symmetry. An approximate (isotropic) treatment of cell e.s.d.'s is used for estimating e.s.d.'s involving l.s. planes.
Refinement. Refinement of F^2^ against ALL reflections. The weighted R-factor wR and goodness of fit S are based on F^2^, conventional R-factors R are based on F, with F set to zero for negative F^2^. The threshold expression of F^2^ > 2sigma(F^2^) is used only for calculating R-factors(gt) etc. and is not relevant to the choice of reflections for refinement. R-factors based on F^2^ are statistically about twice as large as those based on F, and R- factors based on ALL data will be even larger.

### Fractional atomic coordinates and isotropic or equivalent isotropic displacement parameters (Å^2^)

**Table:**

	*x*	*y*	*z*	*U*_iso_*/*U*_eq_	
Ag1	0.0000	0.33644 (2)	0.2500	0.03718 (14)	
P1	0.08413 (5)	0.39847 (5)	0.13087 (7)	0.02910 (19)	
O1	0.06120 (15)	0.17517 (16)	0.3151 (2)	0.0493 (6)	
O2	0.0000	0.0421 (3)	0.2500	0.0783 (13)	
N1	0.0000	0.1292 (3)	0.2500	0.0409 (9)	
C1	0.18506 (17)	0.4287 (2)	0.2384 (3)	0.0342 (7)	
C2	0.2341 (2)	0.5014 (3)	0.2188 (3)	0.0486 (8)	
H2A	0.2160	0.5381	0.1457	0.058*	
C3	0.3100 (2)	0.5200 (3)	0.3075 (4)	0.0635 (11)	
H3A	0.3426	0.5693	0.2943	0.076*	
C4	0.3370 (2)	0.4648 (3)	0.4153 (4)	0.0633 (11)	
H4A	0.3878	0.4771	0.4750	0.076*	
C5	0.2902 (2)	0.3933 (3)	0.4345 (4)	0.0612 (11)	
H5A	0.3094	0.3560	0.5069	0.073*	
C6	0.2142 (2)	0.3747 (3)	0.3484 (3)	0.0472 (8)	
H6A	0.1822	0.3257	0.3639	0.057*	
C7	0.10284 (19)	0.3224 (2)	0.0110 (3)	0.0351 (7)	
C8	0.1568 (2)	0.3472 (3)	−0.0540 (4)	0.0529 (9)	
H8A	0.1849	0.4048	−0.0367	0.064*	
C9	0.1693 (3)	0.2867 (3)	−0.1445 (4)	0.0690 (12)	
H9A	0.2050	0.3040	−0.1888	0.083*	
C10	0.1292 (3)	0.2022 (3)	−0.1684 (4)	0.0702 (13)	
H10A	0.1385	0.1613	−0.2282	0.084*	
C11	0.0753 (3)	0.1761 (3)	−0.1058 (4)	0.0677 (13)	
H11A	0.0477	0.1183	−0.1237	0.081*	
C12	0.0620 (2)	0.2364 (2)	−0.0154 (3)	0.0490 (9)	
H12A	0.0255	0.2189	0.0274	0.059*	
C13	0.04348 (17)	0.5080 (2)	0.0442 (3)	0.0339 (6)	
H13A	0.0432	0.5582	0.1039	0.041*	
H13B	0.0787	0.5278	−0.0046	0.041*	

### Atomic displacement parameters (Å^2^)

**Table:**

	*U*^11^	*U*^22^	*U*^33^	*U*^12^	*U*^13^	*U*^23^
Ag1	0.0364 (2)	0.0378 (2)	0.0391 (2)	0.000	0.01439 (15)	0.000
P1	0.0287 (4)	0.0281 (4)	0.0297 (4)	0.0031 (3)	0.0081 (3)	0.0020 (3)
O1	0.0397 (13)	0.0446 (14)	0.0527 (14)	0.0038 (10)	−0.0010 (11)	0.0004 (11)
O2	0.077 (3)	0.038 (2)	0.118 (4)	0.000	0.029 (3)	0.000
N1	0.042 (2)	0.033 (2)	0.049 (2)	0.000	0.0154 (19)	0.000
C1	0.0290 (15)	0.0384 (16)	0.0348 (15)	0.0051 (12)	0.0096 (13)	−0.0052 (13)
C2	0.0403 (19)	0.049 (2)	0.054 (2)	−0.0047 (15)	0.0113 (16)	−0.0005 (16)
C3	0.0357 (19)	0.063 (3)	0.088 (3)	−0.0127 (17)	0.014 (2)	−0.018 (2)
C4	0.0361 (19)	0.082 (3)	0.059 (2)	0.006 (2)	−0.0046 (18)	−0.028 (2)
C5	0.047 (2)	0.083 (3)	0.043 (2)	0.015 (2)	−0.0008 (18)	−0.0020 (19)
C6	0.0372 (18)	0.062 (2)	0.0404 (18)	0.0085 (16)	0.0087 (15)	0.0075 (16)
C7	0.0380 (17)	0.0331 (16)	0.0326 (15)	0.0098 (12)	0.0088 (13)	−0.0003 (12)
C8	0.054 (2)	0.052 (2)	0.060 (2)	−0.0011 (17)	0.0288 (19)	−0.0087 (17)
C9	0.077 (3)	0.081 (3)	0.062 (3)	0.013 (2)	0.040 (2)	−0.015 (2)
C10	0.097 (3)	0.063 (3)	0.049 (2)	0.027 (2)	0.021 (2)	−0.014 (2)
C11	0.107 (4)	0.040 (2)	0.047 (2)	0.000 (2)	0.011 (2)	−0.0088 (17)
C12	0.071 (2)	0.0376 (18)	0.0369 (17)	−0.0022 (16)	0.0145 (17)	0.0004 (14)
C13	0.0335 (16)	0.0294 (15)	0.0366 (15)	0.0031 (12)	0.0080 (13)	0.0042 (12)

### Geometric parameters (Å, °)

**Table:**

Ag1—P1	2.4066 (9)	C5—C6	1.376 (5)
Ag1—P1^i^	2.4066 (9)	C5—H5A	0.9300
Ag1—O1^i^	2.508 (2)	C6—H6A	0.9300
Ag1—O1	2.508 (2)	C7—C12	1.381 (5)
P1—C7	1.815 (3)	C7—C8	1.386 (5)
P1—C1	1.816 (3)	C8—C9	1.385 (5)
P1—C13	1.834 (3)	C8—H8A	0.9300
O1—N1	1.250 (3)	C9—C10	1.357 (7)
O2—N1	1.225 (5)	C9—H9A	0.9300
N1—O1^i^	1.250 (3)	C10—C11	1.370 (7)
C1—C2	1.382 (5)	C10—H10A	0.9300
C1—C6	1.393 (4)	C11—C12	1.387 (5)
C2—C3	1.388 (5)	C11—H11A	0.9300
C2—H2A	0.9300	C12—H12A	0.9300
C3—C4	1.381 (6)	C13—C13^ii^	1.521 (6)
C3—H3A	0.9300	C13—H13A	0.9700
C4—C5	1.343 (6)	C13—H13B	0.9700
C4—H4A	0.9300		
			
P1—Ag1—P1^i^	137.49 (4)	C4—C5—H5A	119.6
P1—Ag1—O1^i^	115.92 (7)	C6—C5—H5A	119.6
P1^i^—Ag1—O1^i^	102.63 (7)	C5—C6—C1	120.4 (4)
P1—Ag1—O1	102.63 (6)	C5—C6—H6A	119.8
P1^i^—Ag1—O1	115.92 (7)	C1—C6—H6A	119.8
O1^i^—Ag1—O1	50.52 (11)	C12—C7—C8	119.1 (3)
C7—P1—C1	105.68 (14)	C12—C7—P1	118.6 (3)
C7—P1—C13	103.59 (14)	C8—C7—P1	122.4 (3)
C1—P1—C13	105.93 (14)	C9—C8—C7	120.3 (4)
C7—P1—Ag1	117.71 (11)	C9—C8—H8A	119.8
C1—P1—Ag1	109.46 (10)	C7—C8—H8A	119.8
C13—P1—Ag1	113.56 (10)	C10—C9—C8	119.8 (4)
N1—O1—Ag1	95.88 (19)	C10—C9—H9A	120.1
O2—N1—O1	121.14 (18)	C8—C9—H9A	120.1
O2—N1—O1^i^	121.14 (18)	C9—C10—C11	121.0 (4)
O1—N1—O1^i^	117.7 (4)	C9—C10—H10A	119.5
C2—C1—C6	118.3 (3)	C11—C10—H10A	119.5
C2—C1—P1	124.7 (2)	C10—C11—C12	119.7 (4)
C6—C1—P1	117.0 (3)	C10—C11—H11A	120.2
C1—C2—C3	120.5 (4)	C12—C11—H11A	120.2
C1—C2—H2A	119.8	C7—C12—C11	120.1 (4)
C3—C2—H2A	119.8	C7—C12—H12A	119.9
C4—C3—C2	119.6 (4)	C11—C12—H12A	119.9
C4—C3—H3A	120.2	C13^ii^—C13—P1	110.4 (3)
C2—C3—H3A	120.2	C13^ii^—C13—H13A	109.6
C5—C4—C3	120.4 (3)	P1—C13—H13A	109.6
C5—C4—H4A	119.8	C13^ii^—C13—H13B	109.6
C3—C4—H4A	119.8	P1—C13—H13B	109.6
C4—C5—C6	120.9 (4)	H13A—C13—H13B	108.1

Symmetry codes: (i) −*x*, *y*, −*z*+1/2; (ii) −*x*, −*y*+1, −*z*.
